# Improve the satisfaction of medical staff on the use of home nursing mobile APP by using a hybrid multi-standard decision model

**DOI:** 10.1186/s12912-024-01918-9

**Published:** 2024-05-09

**Authors:** Ziwei Ke, Weiyang Qian, Nan Wang, Yen-Ching Chuang, Biying Wei, Jing Feng

**Affiliations:** 1 School of Nursing, Zhejiang Pharmaceutical University, Ningbo, Zhejiang China; 2 Neurosurgery, Taizhou Hospital of Zhejiang Province Affiliated to Wenzhou Medical University, Linhai, Zhejiang China; 3 Intensive Care Unit, Taizhou Hospital of Zhejiang Province Affiliated to Wenzhou Medical University, Zhejiang Linhai, China; 4 Business College, Taizhou University, 318000 Taizhou, Zhejiang China; 5 Institute of Public Health & Emergency Management, Taizhou University, 318000 Taizhou, Zhejiang China; 6 Key Laboratory of evidence-based Radiology of Taizhou, 317000 Linhai, Zhejiang China; 7 Shenzhen Third People’s Hospital, 518112 Shenzhen, Guangdong China; 8 Nursing Department, Taizhou Hospital of Zhejiang Province Affiliated to Wenzhou Medical University, Linhai, Zhejiang China

**Keywords:** Home nursing, Mobile Application (APP), Satisfaction, Decision making and trial evaluation laboratory (DEMATEL), Importance-performance analysis (IPA), Multi-criteria decision-making (MCDM)

## Abstract

**Objective:**

To identify critical satisfaction gaps in a home nursing mobile application (APP) using a systematic decision-making model.

**Methods:**

Initially, the decision-making trial and evaluation laboratory method was used to analyze the relationship structure and corresponding weights among the indicators. The Importance-Performance Analysis (IPA) method was used to identify the categories of all indicators and their corresponding strategic directions. Twenty-six home nursing specialists currently providing home nursing services were recruited for this study.

**Results:**

The IPA results revealed that “Assurance,” “Reliability,” and “Personal security protection” are critical satisfaction gaps. From the influence network and weight results, “information quality” and “system quality” were the critical quality factors in the home nursing mobile APP. The influence of the network relationship structure and weight demonstrated a 98.12% significance level, indicating good stability.

**Conclusion:**

Continuous improvement in information and system quality is recommended to optimize the overall quality of the home nursing mobile APP. Additionally, user demands should be considered, and personal safety guarantee functions should be developed and integrated into the system to ensure the safety of home nursing workers.

## Introduction

With the rapid development of human society, population aging has become a global social issue [1]. According to the National Bureau of Statistics, 720 million people aged 65 years or older, constituting 9.32% of the total population, in 2019. By the end of 2020, there were 99 aging countries and regions across the globe [2]. Additionally, it is estimated that 8% and 10% of individuals aged 65 and above are home-bound, facing challenges in accessing healthcare owing to physical, psychiatric, or social limitations [3].. Home nursing, a service provided by medical professionals, offers essential health and personal care support, enabling people to remain in their homes rather than opting for institutional care. This approach has improved patient and caregiver experiences and reduced healthcare costs, making it an effective strategy for governments addressing aging-related challenges [4]. The outbreak of COVID-19 has further emphasized the importance of home nursing, particularly for older people and those with chronic conditions who are at a heightened risk of severe illness [5, 6].

Recently, the rapid development of mobile device technology has given rise to many internet-based health service systems, including internet-based home nursing. This new service model involves medical workers providing health services at patients’ homes after obtaining orders via the Internet [7]. Compared with the traditional model, Internet-based home nursing conserves human resources while ensuring accurate, real-time, and highly efficient delivery of services, meeting individuals’ healthcare demands [8, 9]. In December 2020, the National Health Commission of China issued a notice to guide and enforce the development of Internet-based home nursing programs, recognizing the transformative potential of this approach.

The system’s effectiveness is fundamental to ensuring Internet-based home nursing care services. Poor system design can lead to inefficiency and errors, resulting in user frustration, dissatisfaction, and eventual rejection. Conversely, a perfect and pertinent system can reduce users’ cognitive and physical demands, enhancing their efficiency and productivity [10]. Therefore, it is necessary to identify critical satisfaction gaps in the home nursing mobile APP and formulate strategies for further improvement.

While previous research on the home nursing mobile APP has primarily focused on usage effects, behavior, and influencing factors [11], studies focusing on improving system effectiveness from the perspective of end users are scarce. Since doctors and nurses are the frontline users of the home nursing mobile APP, its effectiveness directly influences their work, and any hesitancy or reluctance on their part is an important factor affecting the system’s implementation [12]. Consequently, this study aimed to bridge the existing gap by identifying critical satisfaction gaps in a home nursing mobile APP from the perspective of these end users.

However, to achieve this goal, Multicriteria Decision-Making (MCDM) was employed. It is a modern scientific method that facilitates the evaluation, selection, and improvement of alternatives based on a system that incorporates both qualitative and quantitative factors [13]. It assists experts and managers in simplifying decision-making processes by systematically evaluating these factors [14]. Furthermore, its wide application in management is well-documented [15–18]. By incorporating the practical experience of experts and end users, this study seeks to improve the accuracy of standard assessments and help identify the most influential criteria in their respective fields.

## Materials and methods

### Study design and modeling process

**Fig. 1:**
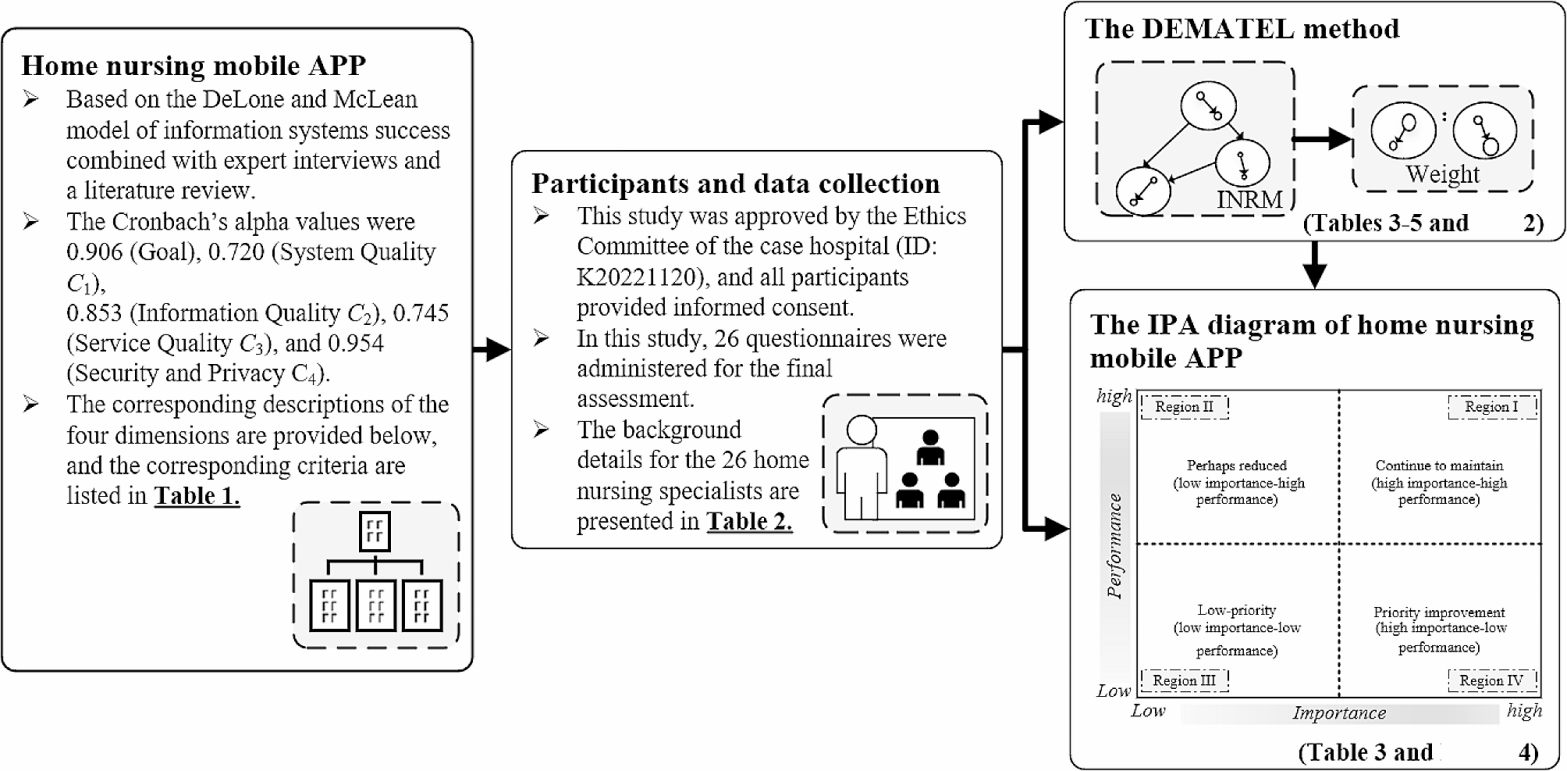
The design and modeling process of this study

This study employed a descriptive questionnaire design. The decision model comprised three components. First, based on previous studies and in-depth discussions with experts, the key elements affecting the effectiveness of the home nursing mobile APP were identified. Subsequently, the Decision-Making Trial and Evaluation Laboratory (DEMATEL) technique was used to evaluate the weightings of the indicators, and an influential network-relation map (INRM) was used to determine the source influencing factors. Finally, the Importance-Performance Analysis (IPA) method was used to analyze the overall situation related to the system effectiveness criteria and corresponding satisfaction with the home nursing mobile APP. The design and modeling process of this study is shown in Fig. 1.

### Home nursing mobile APP

Based on the DeLone and McLean model of information systems success, combined with expert interviews and a literature review [19–27], an effective evaluation system for the home nursing mobile APP was developed. The Cronbach’s alpha values were 0.906 (goal), 0.720 (System Quality *C*_1_), 0.853 (Information Quality *C*_2_), 0.745 (Service Quality *C*_3_), and 0.954 (Security and Privacy *C*_4_). These values signify the robust reliability of the evaluation model, making it suitable for application in this study. The descriptions of the four dimensions and the corresponding criteria are provided below and listed in Table 1.

**Table 1:** Satisfaction evaluation framework of mobile home nursing service system (Cronbach’s Alpha = 0.906)

Dimension/Criteria	Indicator meaning	Ref
System Quality (*C*_1_) Alpha = 0.720	System operation quality of users using home care APP platform	
Attractiveness (*C*_11_)	The interface of the APP platform is beautifully designed, with consistent overall appearance and beautiful color matching.	[19]
Learnability (*C*_12_)	Users initially use the functions of an easy-to-learn APP platform	[20]
Operability (*C*_13_)	Users can easily operate the functions of APP platform	[20]
Integration (*C*_14_)	The functions of the APP platform have good integration	[21]
Stability (*C*_15_)	The APP platform runs very stably.	[22]
Information quality (*C*_2_) Alpha = 0.853	Information Quality Output from the APP Platform	
Accuracy (*C*_21_)	The information provided by the APP platform is consistent with the actual situation, and there is no error information.	[22, 23]
Comprehensive (*C*_22_)	The information provided by the APP platform is comprehensive and can meet the information types and contents required by home care businesses.	[19, 22]
Availability (*C*_23_)	The information provided by the APP platform is valuable for home care services and can be used.	[23]
Continuity (*C*_24_)	The information provided by APP platform is continuous and dynamic, which can reflect the long-term changes of patients’ conditions.	[23]
Accessibility (*C*_25_)	The APP platform can provide relevant information in time.	[19, 23]
Service quality (*C*_3_) Alpha = 0.745	The quality-of-service users get from APP platform developers and maintainers.	
Assurance (*C*_31_)	The APP platform has all the functions needed to provide home care services and has a continuously updated operation mechanism.	[20]
Empathy (*C*_32_)	The APP platform developers/maintenance personnel will regularly understand the specific needs of users.	[23]
Reliability (*C*_33_)	APP platform developers/maintenance personnel can solve the problems encountered by users when using the platform as soon as possible.	[20, 23]
Customizable (*C*_34_)	The APP platform allows users to make personalized settings (such as message push and reminder settings) according to their personal characteristics/preferences.	[24]
Safety and security (*C*_4_) Alpha = 0.954	Users can get corresponding guarantee services when using APP platform functions.	
Privacy protection (*C*_41_)	Without the user’s consent, the APP platform will not share any data of the user with other parties.	[25]
Personal security protection (*C*_42_)	APP platform monitors the action track of nursing staff in real-time and has a one-button alarm function to ensure the personal safety of nursing staff.	[26]
Payment security (*C*_43_)	The APP platform ensures the safety of the payment process for patients/family members.	[27]

### Participants and data collection

This study was approved by the Ethics Committee of the case hospital (ID: K20221120), and all participants provided informed consent. Data collection occurred between December 2022 and January 2023. Participants, specifically doctors and nurses currently providing home nursing services, were recruited through referrals from the department head. They were informed about the study’s objectives and assured of its harmlessness, after which they provided written consent and completed anonymous questionnaires. Previous studies indicate that the typical sample size for relevant studies using MCDM approaches ranged between 10 and 30 specialists [28]. In this study, 26 questionnaires were administered for the final assessment. The background details for the 26 home nursing specialists are presented in Table 2.

**Table 2:** The background description of 26 home nursing specialist

Characteristics	Value (%)
Sex	
Male	5 (19.2%)
Female	21(80.8%)
Age	
30–39	14(53.8%)
40–49	9(34.6%)
50–59	3(11.5%)
Education	
Technical secondary school	1(3.8%)
Junior college	1(3.8%)
Undergraduate	21(80.8%)
Master and above	3(11.5%)
Years of work experience in home nursing services	
Under 3 years	10(38.4%)
3–5	6(23.0%)
5 and above	10(38.4%)
Professional title	
Nurse	23(88.5%)
Doctor	3(11.5%)

### The DEMATEL method

DEMATEL is an analytical technique designed to aid decision-making in complex scenarios [29]. It simplifies the structure of multifactorial systems and identifies key influencing factors [30, 31]. Based on graph theory, DEMATEL supports the development of knowledge and experience by analyzing the logical correlations and direct influence relationships between factors in complex systems, revealing key factors [32]. Due to its universal and simple mechanism, DEMATEL has attracted the attention of many scholars across various disciplines, including environmental science, management, engineering, medicine, and nursing [33–37]. While detailed information regarding the DEMATEL method can be found in related sources [32, 33, 37, 38], the brief calculation steps are as follows:

Step 1: According to the practical experience of participants and based on a set of Likert five-point scales [i.e., ranging from no impact (0) to very large impact (4)], the degree of mutual influence among all indicators was documented, and subsequently integrated into an initial influence matrix (***B***) of group opinions using the average method, as shown in Eq. (1).

1 $${\varvec{B}}={\left[ {\begin{array}{*{20}{c}} {{b_{11}}}& \cdots &{{b_{1j}}}& \cdots &{{b_{1n}}} \\ \vdots & \ddots & \vdots & \ddots & \vdots \\ {{b_{i1}}}& \cdots &{{b_{ij}}}& \cdots &{{b_{in}}} \\ \vdots & \ddots & \vdots & \ddots & \vdots \\ {{b_{n1}}}& \cdots &{{b_{nj}}}& \cdots &{{b_{nn}}} \end{array}} \right]_{n \times n}}={\left[ {\frac{{\left( {\sum\nolimits_{{\varphi =1}}^{q} {a_{{ij}}^{\varphi }} } \right)}}{q}} \right]_{n \times n}}$$

where *ij* is the degree to which indicator *i* affects indicator *j*; *n* is the number of indicators; and *q* is the number of respondents.

Step 2: The initial influence matrix (***B***) was converted into a normalized proportional influence matrix (***E***) based on the correlation formula, as shown in Eqs. (2) and (3):

2 $$\Upsilon =\hbox{max} \left\{ {\hbox{max} \sum\nolimits_{{j=1}}^{n} {{b_{ij}}},\hbox{max} \sum\nolimits_{{i=1}}^{n} {{b_{ij}}} } \right\},i,j \in \left\{ {1,2,...,n} \right\}$$

3 $${\varvec{E}}={{\varvec{B}} \mathord{\left/ {\vphantom {{\varvec{B}} \Upsilon }} \right. \kern-0pt} \Upsilon }$$

Step 3: The normalized proportional influence relation matrix (***E***) was calculated by applying Eq. (4); the total influence relation between indexes was calculated and generated into a matrix (***T***).

4 $${\varvec{T}}={\varvec{E}}+{{\varvec{E}}^2}+...+{{\varvec{E}}^\eta }={\varvec{E}}{({\varvec{I}} - {\varvec{E}})^{ - 1}},{\text{when}}{\text{ }}\mathop {\lim }\limits_{{\eta \to \infty }} {{\varvec{E}}^\eta }={\left[ 0 \right]_{n \times n}}$$

Step 4: Based on the viewpoints of influence and affected, four influence indicators were produced from the total influence relation matrix: influence, affected, influence centrality, and influence causality, as shown in Eqs. (5)–(8):

5 $${\rm{Influence:}}\,{o_i} = \left( {{o_1},{o_2}, \ldots,{o_n}} \right) = {\left[ {\sum\nolimits_{j = 1}^n {{t_{ij}}} } \right]_{n \times 1}}$$

6 $${\rm{Affected:}}\,{u_i} = \left( {{u_1},{u_2}, \ldots,{u_n}} \right) = \left( {{t_j}} \right)_{1 \times n}^\Gamma = \left[ {\sum\nolimits_{i = 1}^n {{t_{ij}}} } \right]_{1 \times n}^\Gamma $$

7 $$ {\rm{Influence}}\,{\rm{centrality:}}\,{o_i} + {u_i} $$

8 $$ {\rm{Influence}}\,{\rm{causality:}}\,{o_i} - {u_i} $$

A positive ${o_i} - {u_i}$ value indicates significant influence property, referred to as “Cause”; conversely, a negative value signifies significant affected property, referred to as “Effect.”

Step 5: Influence centrality was converted into influence weight, as shown in Eq. (9).

9 $${w_i}={{{o_i}+{u_i}} \mathord{\left/ {\vphantom {{{o_i}+{u_i}} {\sum\nolimits_{{i=1}}^{n} {({o_i}+{u_i})} }}} \right. \kern-0pt} {\sum\nolimits_{{i=1}}^{n} {({o_i}+{u_i})} }}$$

The larger the weight value, the stronger the influence intensity of the index in the entire evaluation index system, defining it as a key influencing factor.

### The IPA method

The IPA method, introduced by Martilla and James, constitutes a fundamental decision-analysis technology [39]. It involves comparing or classifying attributes based on their relative importance and performance ratings, aiding decision-makers in prioritizing areas for improvement [40]. Owing to its simplicity and ease of use, the IPA method finds widespread application in public health, nursing, and education [16, 41–43]. The application steps of the IPA method are as follows [44]:

Step 1: Participants used the Likert five-point scale [i.e., very dissatisfied (1) to very satisfied (5)] to evaluate their satisfaction with all indicators within the home nursing mobile APP.

Step 2: The x-axis and y-axis of the IPA chart represent the weight and satisfaction of the DEMATEL, respectively. The average value serves as the central value to distinguish the relative regional categories of all the indicators.

Step 3: Decision-making schemes for the indicators in the four regions were proposed, including:

(i) Continue to maintain (high importance-high performance): decision-makers should continue to invest resources to maintain the current performance as it represents a key competitive advantage.
(ii) Perhaps reduced (low importance-high performance): policymakers should reduce resource input to prevent waste or inefficient resource utilization.
(iii) Low-priority (low importance-low performance) policymakers should temporarily withhold resources to improve their satisfaction with these indicators.
(iv) Priority improvement (high importance-low performance): policymakers should prioritize resources to improve current satisfaction performance because this is a critical satisfaction gap.

### Ethical approval

All procedures were conducted according to the guidelines of our institution’s ethics committee and adhered to the principles of the Declaration of Helsinki. Participant data were anonymized to ensure confidentiality. The Institutional Review Board of Taizhou Hospital of Zhejiang Province (ID: K20221120) approved the informed consent procedure for this study and the entire study.

## Results

### Participants’ characteristics

In this study, 80.8% (*n* = 21) of the respondents were female, and 88.5% (*n* = 23) were nurses, with over 50% having more than 3 years of experience in home nursing. In addition, most respondents had a bachelor’s degree (80.8%). Details of the respondents are shown in Table 2.

### DEMATEL results for composite indicators and weights

**Fig. 2:**
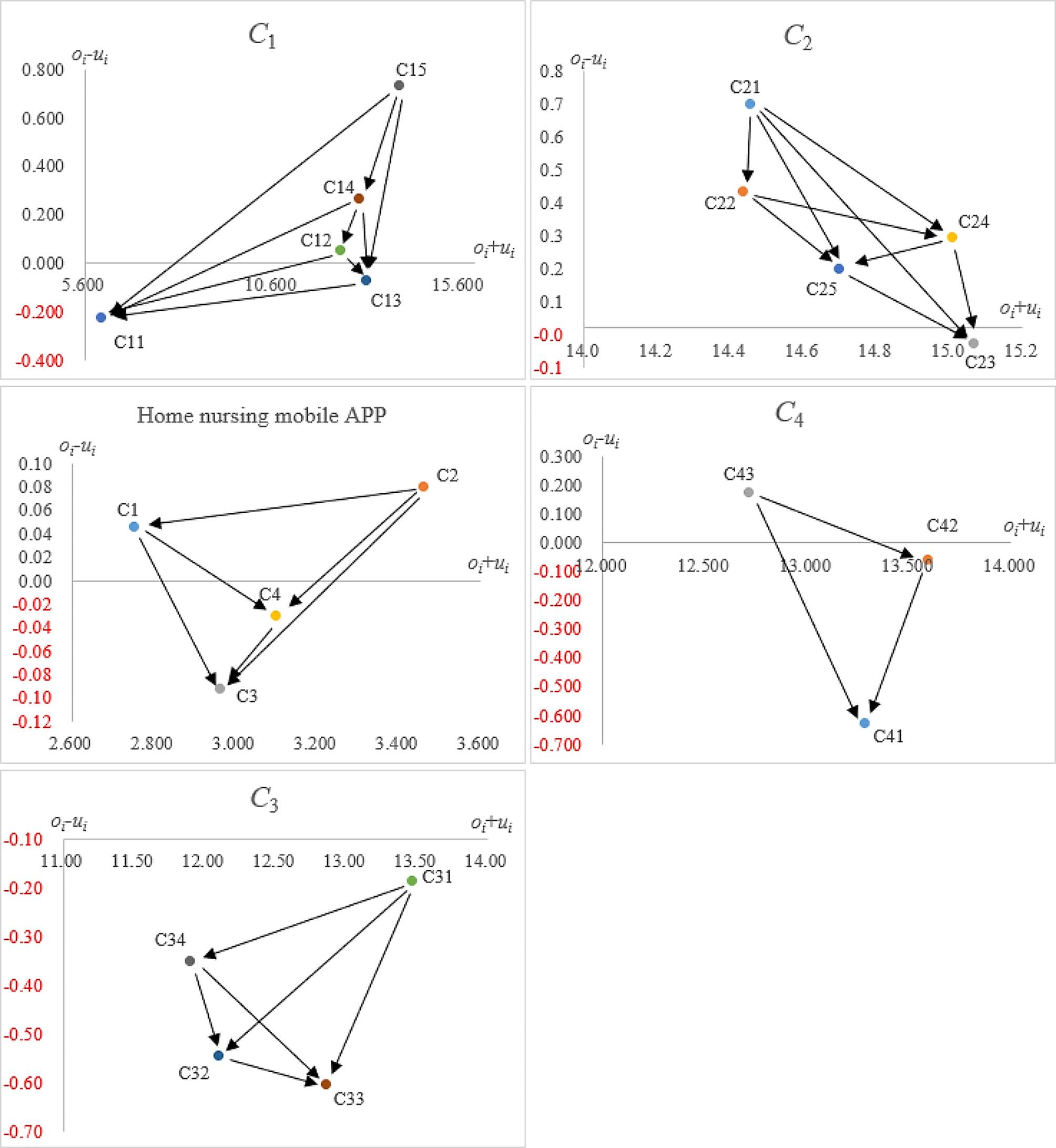
Influential network-relation map (INRM)

The initial influence matrix (***B***) represented the degree of interaction between indicators as assessed by all participants based on practical experience, as shown in Table 3. The statistical significance of matrix (***B***) was 98.12%, with a gap error of 1.82%, indicating a high confidence level in this dataset. Matrix (***B***) yielded four influence indexes for all indicators using Eqs. (2)–(8), as shown in Table 4 and Fig. 2.

**Table 3:** Initial influence matrix ***B***

	*C*_11_	*C*_12_	*C*_13_	*C*_14_	*C*_15_	*C*_21_	*C*_22_	*C*_23_	*C*_24_	*C*_25_	*C*_31_	*C*_32_	*C*_33_	*C*_34_	*C*_41_	*C*_42_	*C*_43_
*C*_11_	0.000	1.231	1.500	1.385	0.692	0.654	0.808	1.154	0.846	0.692	0.731	0.731	0.615	0.962	1.077	0.808	0.500
*C*_12_	1.192	0.000	3.038	1.923	1.808	2.038	1.808	2.500	2.077	1.923	1.808	1.769	1.808	1.538	1.885	1.846	2.115
*C*_13_	1.115	3.308	0.000	1.962	1.962	2.423	1.885	2.423	2.308	2.346	1.692	1.654	1.654	1.692	2.115	2.115	1.808
*C*_14_	1.308	2.115	2.538	0.000	2.346	2.346	2.385	2.462	2.423	2.154	1.577	1.500	1.923	1.577	2.154	2.192	1.769
*C*_15_	0.885	1.846	2.423	2.538	0.000	2.731	2.731	2.615	2.923	2.615	2.346	1.808	2.192	1.885	2.462	2.308	2.269
*C*_21_	1.038	1.923	2.192	2.269	2.615	0.000	2.846	2.962	2.962	3.000	2.577	2.038	2.192	1.808	2.308	2.462	2.385
*C*_22_	1.077	2.000	2.192	2.846	2.654	2.462	0.000	2.846	2.923	2.538	2.577	2.038	2.308	2.192	2.308	2.038	2.000
*C*_23_	0.962	2.615	2.385	2.385	2.423	2.692	2.923	0.000	2.885	2.808	2.308	2.115	2.423	2.269	2.346	2.115	1.769
*C*_24_	1.154	2.269	2.269	2.500	2.500	2.538	2.923	3.000	0.000	2.846	2.654	2.115	2.269	2.269	2.500	2.308	2.077
*C*_25_	1.000	2.154	2.269	2.308	2.462	2.846	2.731	2.923	3.038	0.000	2.192	2.038	2.192	2.115	2.269	2.192	2.231
*C*_31_	0.808	1.808	2.000	1.808	1.885	2.308	2.154	2.308	2.308	2.231	0.000	2.269	2.423	2.269	2.269	2.192	2.000
*C*_32_	0.923	1.615	1.500	1.615	1.731	1.808	1.923	2.000	1.654	2.000	1.846	0.000	2.077	2.038	2.000	2.154	1.808
*C*_33_	0.846	1.538	1.769	1.731	1.923	1.769	1.885	2.038	2.115	2.192	2.115	2.000	0.000	2.192	2.269	2.231	1.808
*C*_34_	1.231	1.846	1.846	1.692	1.846	1.615	1.615	1.885	1.923	1.962	2.308	2.077	2.000	0.000	1.808	1.615	1.577
*C*_41_	0.769	1.538	1.731	1.615	1.885	1.885	1.846	2.192	1.962	2.000	2.269	2.346	2.423	1.577	0.000	2.846	2.538
*C*_42_	0.692	1.500	1.846	1.500	2.077	2.077	2.231	2.346	2.192	2.577	2.692	2.577	2.500	2.077	2.423	0.000	2.308
*C*_43_	0.500	1.538	1.692	1.500	2.115	1.923	2.077	2.231	2.154	2.192	2.192	2.269	2.385	1.923	2.577	2.577	0.000

Note: The significant confidence equation is$((\sum\nolimits_{{i=1}}^{n} {\sum\nolimits_{{j=1}}^{n} {\left| {b_{{ij}}^{q} - b_{{ij}}^{{q - 1}}} \right|} } /b_{{ij}}^{q})/(n(n - 1))) \times 100\% =1.82\% <5\%$, i.e., significant confidence is 98.12%

**Table 4:** The result of four influence indicators for dimensions and criteria

	*o*_*i*_	*u*_*i*_	*o*_*i*_+*u*_*i*_	*o*_*i*_-*u*_*i*_		*o*_*i*_	*u*_*i*_	*o*_*i*_+*u*_*i*_	*o*_*i*_-*u*_*i*_
*C*_1_	1.399	1.354	2.753	0.044	*C*_11_	2.913	3.142	6.054	-0.229
					*C*_12_	6.244	6.193	12.437	0.051
					*C*_13_	6.531	6.606	13.137	-0.075
					*C*_14_	6.600	6.336	12.936	0.264
					*C*_15_	7.378	6.652	14.030	0.727
*C*_2_	1.772	1.692	3.464	0.079	*C*_21_	7.565	6.892	14.457	0.673
					*C*_22_	7.423	7.014	14.438	0.409
					*C*_23_	7.510	7.559	15.068	-0.049
					*C*_24_	7.640	7.369	15.009	0.271
					*C*_25_	7.438	7.262	14.700	0.176
*C*_3_	1.435	1.528	2.963	-0.093	*C*_31_	6.644	6.832	13.475	-0.188
					*C*_32_	5.782	6.327	12.109	-0.545
					*C*_33_	6.132	6.737	12.869	-0.605
					*C*_34_	5.776	6.130	11.905	-0.354
*C*_4_	1.535	1.566	3.101	-0.031	*C*_41_	6.327	6.959	13.286	-0.632
					*C*_42_	6.766	6.830	13.596	-0.065
					*C*_43_	6.444	6.273	12.717	0.171

All indicators could be further categorized into dimensions and criterion levels. First, from the “Influence causality (${o_i} - {u_i}$)” index analysis, “(*C*_1_)” and “(*C*_2_)” were identified as cause indicators, mainly affecting other dimensions. “(*C*_3_)” and “(*C*_4_)” were identified as effectiveness indicators, mainly affected by other dimensions. Subsequently, the “Influence centrality (${o_i}+{u_i}$)” index analysis revealed a ranking from high to low as “(*C*_2_),” “(*C*_4_)”, “(*C*_3_)” and “(*C*_1_).”

The impact weights of all dimensions and the corresponding criteria are listed in Table 5. From the dimension level, “(*C*_2_)” had the highest weight. At the criteria level, “(*C*_15_),” “(*C*_23_),” “ (*C*_31_)” and “ (*C*_42_)” were the top standard with the highest weight in each dimension.

**Table 5:** The results of influential weights for dimensions and criteria

	Local weight	Rank		Local weight	Rank	Global weight	Rank
*C*_1_	0.224	4	*C*_11_	0.103	5	0.023	17
			*C*_12_	0.212	4	0.048	16
			*C*_13_	0.224	2	0.050	14
			*C*_14_	0.221	3	0.049	15
			*C*_15_	0.239	1	0.054	13
*C*_2_	0.282	1	*C*_21_	0.196	4	0.055	11
			*C*_22_	0.196	5	0.055	12
			*C*_23_	0.205	1	0.058	7
			*C*_24_	0.204	2	0.057	8
			*C*_25_	0.200	3	0.056	10
*C*_3_	0.241	3	*C*_31_	0.268	1	0.065	4
			*C*_32_	0.240	3	0.058	6
			*C*_33_	0.256	2	0.062	5
			*C*_34_	0.236	4	0.057	9
*C*_4_	0.252	2	*C*_41_	0.336	2	0.085	2
			*C*_42_	0.343	1	0.087	1
			*C*_43_	0.321	3	0.081	3

### The IPA results of four regions

**Fig. 3:**
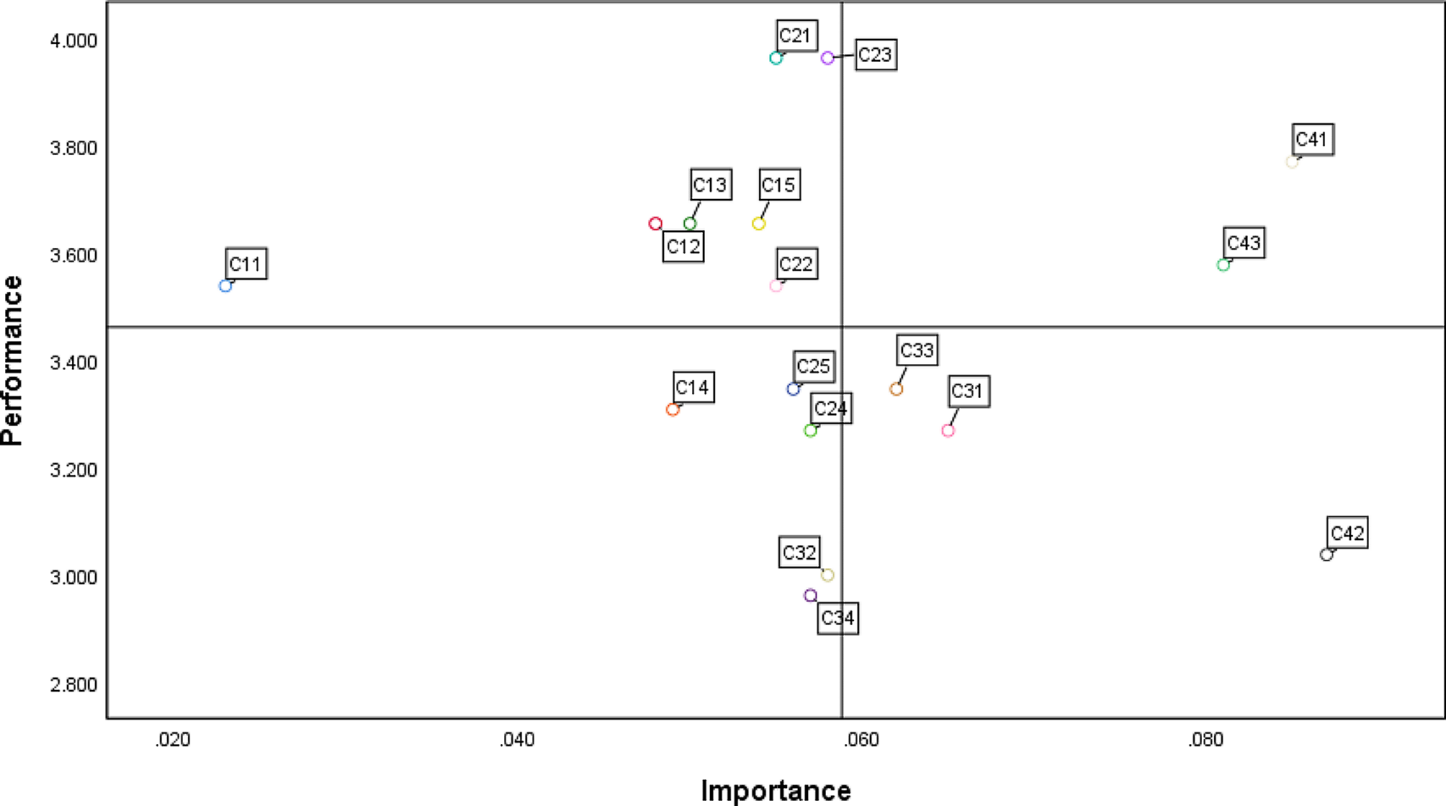
The IPA diagram of mobile home nursing service system

The IPA results are presented in Table 6 and Fig. 3. “Payment security” (*C*_43_), and “Privacy protection” (*C*_41_) indicators fell into the (I) Keep region. “Attractiveness” (*C*_11_), “Learnability” (*C*_12_), “Operability” (*C*_13_), “Stability” (*C*_15_), “Accuracy” (*C*_21_), “Comprehensive” (*C*_22_), “Availability” (*C*_23_) fell into the (II) Reduce region. “Integration” (*C*_14_), “Continuity” (*C*_24_), “Accessibility” (*C*_25_), “Empathy” (*C*_32_), “Customizable” (*C*_34_) fell into the (III) Low Priority region. “Assurance” (*C*_31_), “Reliability” (*C*_33_), and “Personal security protection” (*C*_42_) fell into the (IV) Improve region.

**Table 6:** IPA results

	Importance	Performance	Region
Attractiveness (*C*_11_)	0.023	3.538	II
Learnability (*C*_12_)	0.048	3.654	II
Operability (*C*_13_)	0.050	3.654	II
Integration (*C*_14_)	0.049	3.308	III
Stability (*C*_15_)	0.054	3.654	II
Accuracy (*C*_21_)	0.055	3.962	II
Comprehensive (*C*_22_)	0.055	3.538	II
Availability (*C*_23_)	0.058	3.962	II
Continuity (*C*_24_)	0.057	3.269	III
Accessibility (*C*_25_)	0.056	3.346	III
Assurance (*C*_31_)	0.065	3.269	IV
Empathy (*C*_32_)	0.058	3.000	III
Reliability (*C*_33_)	0.062	3.346	IV
Customizable (*C*_34_)	0.057	2.962	III
Privacy protection (*C*_41_)	0.085	3.769	I
Personal security protection (*C*_42_)	0.087	3.038	IV
Payment security (*C*_43_)	0.081	3.577	I
Mean	0.058	3.461	

## Discussions

This study established a hybrid multi-standard decision model aimed at identifying the factors influencing the satisfaction of medical staff with the use of home nursing mobile APP. The main findings revealed the causality relationship between each factor and the weight of each factor, allowing the identification of priority factors in the development of the home nursing mobile APP. In this discussion, these findings were analyzed, and systematic improvement directions were proposed to provide a decision-making basis for improving user satisfaction.

### Source influencing factors from INRM

In this study, the Influential Network-Relation Map (Fig. 2) was used to explore the source influencing factors for the effectiveness of a home nursing mobile APP. At the dimension level, the cause factors are “Information Quality” (*C*_2_) and “System Quality” (*C*_1_), showing that these factors are more inclined to affect other factors. This indicates that system and information quality are the basic components of an APP and should be primarily considered during APP building, which aligns with Zhang [45]. Notably, “Information Quality” (*C*_*2*_) was found to have an overall effect on improving the effectiveness of home nursing mobile APP. According to Kim, Chang [46], a home nursing mobile APP is a service system serving as the primary channel for interactive communication between service providers and end-users. This implies that service system interactivity is initiated by the user, prompting the service providers to perform a service; the most important step of this process is the interaction of information between them [47]. Consequently, information quality is particularly important for work efficiency and improving the overall quality of home nursing services, which is also fundamental for a home nursing mobile APP.

Similarly, the same method was used to analyze the dependence and influence relationships among the criteria in each dimension. In the “System Quality” (*C*_1_) criteria, “Stability” (*C*_15_) was the main influence criterion. Stability refers to fewer accidents in the process of use to ensure the normal operation of the software [45]. Therefore, it should be the first factor to consider in APP construction. In the “Information Quality” (*C*_2_) criteria, “Accuracy” (*C*_21_) was the main influence criterion. This indicates that improving information accuracy is the first step toward enhancing its quality and availability. Interestingly, “Assurance” (*C*_31_) exhibited the highest impact in “Service Quality” (*C*_3_). This reveals that, in the service quality dimension, the functionality of the home nursing mobile APP is crucial. An APP equipped with all necessary functions, and a continuously updated operational mechanism, fosters reliability and empathy among users, thereby comprehensively elevating service quality [48]. Finally, within the “Security and Privacy” (*C*_4_) criteria, “Payment security” (*C*_43_) exhibited the most significant impact. This may be related to the online payment process involving a large amount of personal information from users directly related to privacy, safety, and personal security [49]. According to a national survey, concerns about security and privacy ranked among the common reasons for APPs avoidance. This observation is consistent with the findings of the present study. All results advocate for information security in Internet-based service systems, emphasizing the online payment process [50–52]. Therefore, APPs should provide a refund guarantee, protect passwords and data during payment, and provide a variety of reliable payment channels to ensure the security of the user payment environment [45].

### Key factors from influential weight

Influential weights were calculated to explore the most important factors influencing user satisfaction with using a home nursing mobile APPs. The results showed that at the dimension level, “Information Quality” (*C*_2_) exhibited the highest weight, underscoring its paramount importance in the use of home nursing mobile APP. This finding aligns with previous studies. In home nursing mobile APPs, subpar information quality is directly related to patient insecurity, giving rise to potential medical, legal, and social concerns [51], emphasizing that when services are delivered through smart devices, information quality is a key attribute affecting user satisfaction [47]. Further analysis at the criteria level reveals the following: “Stability” (*C*_15_) was the key factor in“System Quality” (*C*_*1*_). This indicates the importance of creating a reliable and stable user environment, including fewer flashbacks, a low error rate, identifying problems, and repairing vulnerabilities [45]. “Availability” (*C*_23_) was the key factor in “Information Quality” (*C*_2_). This means that the information provided by the APP should be valuable and easy to obtain, thereby significantly improving work efficiency. This is particularly important for doctors and nurses who experience a shortage of human resources and overwhelming workloads [53]. “Assurance” (*C*_31_) was the key factor in “Service Quality” (*C*_3_). For users, an APP should provide all the functions needed, which is the main factor considered when choosing it. Therefore, from the perspective of developers, the development of the APP must be based on a comprehensive investigation of user needs, and it must have a continuously updated operational mechanism, which is the key factor potentialy affecting user loyalty [54]. “Personal security protection” (*C*_42_) exhibited the highest influential weight in “Security & privacy” (*C*_4_). Unlike hospital settings, home nursing involves delivering medical care services in patient’s homes, exposing medical workers to more potential hazards [2]. It is of great significance that a home nursing mobile APP can help ensure the personal security of medical workers during home nursing.

### Implications

In this study, IPA was performed to explore the priority areas for improvement in home nursing mobile APP. The results identified Category IV, including *C*_31_, *C*_33_, and *C*_42_, as the focal points requiring attention. It can be concluded that the primary challenges within home nursing mobile APPs are service quality and security protection. Finally, based on INRM and IPA, a systematic improvement direction is proposed.

According to IPA, improving the service quality of the home nursing mobile APP is a priority. However, the result of INRM revealed that “Service Quality” (*C*_3_) is an effect factor, while “System Quality” (*C*_1_) and “Information Quality” (*C*_2_) are the cause factors more inclined to affect other factors. It indicates that to optimize service quality, enhancing system and information quality remains essential, especially the stability of the system and the accuracy of the information, which were the cause factors in these two dimensions, respectively. The result of INRM also demonstrated that “Assurance” (*C*_31_) was the cause factor in “Service Quality” (*C*_3_). It highlights the importance of investigating user demands, offering unique insights into their perceptions, and guiding the formulation of effective strategies for APP improvement. In the future, detailed investigations into the functional needs of home nursing mobile APPs from the perspective of home nursing service workers are necessary. Furthermore, users should be offered the opportunity to give feedback. This allows problems to be understood more efficiently. It is also crucial for continual updates and improvements to the APP.

Another aspect requiring attention in this study is the personal safety of home nursing workers. In the future, functions designed to ensure the personal safety of medical workers during home nursing, such as real-time positioning systems, whole-process monitoring, one-click calls for help, and safety risk assessment systems, should be integrated into home nursing mobile APPs. Meanwhile, the APP should provide safety training for home nursing workers, including providing relevant information and communication platforms for them, instructing them on the communication process of workplace violence incidents, reporting and emergency response mechanisms, and handling violence incidents.

### Strengthens and limitations of the study

This study leverages academic theory and practical cases to assess the importance of criteria and user satisfaction in the home nursing mobile APP. By identifying critical satisfaction gaps, this study provided actionable recommendations for enhancing this system and, consequently, boosting user intentions to use it. Despite its strengths, this study had some limitations. First, the results were based on a sample of doctors and nurses from a single hospital in China, restricting the breadth of perspectives. Future research should diversify the participant pool to include patients and software developers, enabling a more comprehensive analysis. Additionally, being a case study focused on a home nursing mobile APP, generalizing the findings may be challenging. However, to some extent, this research enriches and improves the theoretical and practical research in the home nursing mobile APP field and provides a new perspective for the home nursing mobile APPs.

## Conclusion

This study revealed that information quality and system quality are the factors influencing home nursing mobile APPs, and the assurance and reliability of service quality and personal security protection are target areas that need further improvement. In the future, information and system quality should be continuously improved, and user demands should be considered. Additionally, personal safety guarantee functions should be developed and integrated into the APP to ensure the safety of home nursing workers.

## Data Availability

The original contributions presented in the study are included in the article materials, further inquiries can be directed to the corresponding authors.
